# Chitosan/PVA Hetero-Composite Hydrogel Containing Antimicrobials, Perfluorocarbon Nanoemulsions, and Growth Factor-Loaded Nanoparticles as a Multifunctional Dressing for Diabetic Wound Healing: Synthesis, Characterization, and In Vitro/In Vivo Evaluation

**DOI:** 10.3390/pharmaceutics14030537

**Published:** 2022-02-28

**Authors:** Yu-Hsiang Lee, Sheng-Jhe Lin

**Affiliations:** 1 Department of Biomedical Sciences and Engineering, National Central University, Taoyuan City 32001, Taiwan; harryzm52020@gmail.com; 2 Department of Chemical and Materials Engineering, National Central University, Taoyuan City 32001, Taiwan

**Keywords:** diabetic wound healing, hydrogel, nanoparticle, perfluorocarbon, oxygen delivery, antibacterial, anti-inflammatory, cell growth

## Abstract

Diabetic foot ulcers remain one of the most difficult-to-treat complications of diabetes and may seriously threaten the life of patients since it frequently results in limb loss due to amputation, suggesting that an effective therapeutic strategy is still urgently needed. In this study, a chitosan-based heterogeneous composite hydrogel encapsulating perfluorocarbon emulsions, epidermal growth factor (EGF)-loaded chitosan nanoparticles, and polyhexamethylene biguanide (PHMB) named P_E_E_NP_PCH was developed for diabetic wound healing. The P_E_E_NP_PCH could sustainably release EGF and PHMB in an ion-rich environment to exert antibacterial effects and promote cell growth for wound repair. In addition, the P_E_E_NP_PCH can provide anti-inflammatory effects functioned by its main constituent of chitosan. Moreover, the P_E_E_NP_PCH can proactively offer oxygen delivery through the incorporation of perfluorocarbon and, therefore, is able to alleviate hypoxia conditions on diabetic wounds. These functionalities enabled a markedly enhanced wound healing efficacy on diabetic rats treated with the P_E_E_NP_PCHs, including thorough re-epithelization, a reduced inflammatory response, faster collagen deposition, and advanced collagen maturation resulting in a 95% of wound closure degree after 15 days that was 12.6% (*p* < 0.05) higher than the value of the group treated with the commercial dressing HeraDerm. Given the aforementioned advantages, together with the known merits of hydrogels, the developed P_E_E_NP_PCH is anticipated to be a feasible tool for clinical diabetic wound treatment.

## 1. Introduction

Diabetic foot ulcer (DFU), one of the most serious diabetic complications, is a progressive wound on diabetic skin with no or a delayed healing rate due to impaired metabolism, immune deficiency, and repeated infections [1]. This health burden may seriously threaten the life of patients since it frequently results in limb loss due to amputation. Amputation is a major sequela of diabetes and occurs approximately every 30 s worldwide [2]. According to statistics, an increasing mortality rate of up to 80% was reported between 90 days and 5 years after amputation in patients with diabetes [3,4], and the survival rate within five years may decrease to approximately 55% and 34% after minor and major amputations due to DFU, respectively [5]. These circumstances together with the increasing global prevalence of diabetes [2], indicate that developing an effective strategy (e.g., on-demand wound dressing) for improved DFU treatment is still urgently needed nowadays.

The main principles for DFU treatment are wound debridement, pressure offloading, revascularization, and infection management. Hydrogels are commonly proposed as a favorable tool for wound repair since they may be able to provide further benefits, including a moist environment, low adhesion, and thermal insulation, to increase the probability of wound healing compared with other dressing forms, such as gauze, bandages, and/or films [6]. However, coverage provided by dressings may form another barrier to oxygen delivery over the exudates on the wound bed, as oxygen plays a crucial role in many biochemical and cellular processes related to wound healing, such as infection control [7,8], construction of the extracellular matrix [9,10], and collagen formation/remodeling [11,12]. Although hyperbaric oxygen therapy (HBOT) and topical pressurized oxygen therapy (TPOT) have been widely utilized to reduce hypoxia for DFU treatment, HBOT is costly, difficult to operate and maintain, toxic to the central nervous system, and may cause barotrauma and/or cell cycle arrest due to overexposure to a high-pressure, high-oxygen environment [13,14], while TPOT is hampered by its complicated and labor-consuming procedures [15].

Perfluorocarbon (PFC) is a fluorine-substituted anthropogenic hydrocarbon that is able to dissolve large volumes of respiratory and other nonpolar gases compared to water [16]. Some PFC liquids, such as perfluorooctyl bromide (PFOB) and perfluorodecalin, are biocompatible and have been widely used in various biochemical and biological applications over the last few decades [17,18], showing great potential for use as oxygen transporters for wound healing. However, PFC is very unlikely to be dispersed in the hydrogel matrix because PFC liquids commonly have a density > 1.5 g/cm^3^ and are virtually insoluble in water.

The inhibition of cell signaling due to dysfunctional physiology in patients with diabetes and the high risk of repeated infections due to the long existence/colocation of massive sloughs and bacteria may further increase the difficulty of diabetic wound healing. Therefore, growth factors and/or cytokines, such as epidermal growth factor (EGF), transforming growth factor β (TGF-β), and platelet-derived growth factor (PDGF) [19], and/or antibacterial agents, such as metal nanoparticles, antibiotics, and/or honey [20], are frequently incorporated into recently developed hydrogels to activate cellular/molecular signaling and exert antimicrobial effects for improved wound healing. However, unfavorable scenarios such as limited drug loading efficiency, generation of detrimental side effects due to overloading of drugs, and/or the occurrence of unpredicted interactions between coloaded reagents [21,22] substantially reduce their therapeutic effects in practical applications.

Nanotechnology may represent a feasible method for the coadministration of PFC, growth factors, and antimicrobial agents in the same hydrogel system since nanocarriers (1) provide a feasible approach to homogeneously disperse each agent in the hydrogel structure, (2) offer increased bioavailability, stability, and security for the payloads, and (3) allow controlled release of the drugs entrapped in the polymeric matrix [23]. In this study, we sought to design, fabricate and explore a new type of chitosan-based heterogeneous composite hydrogel containing PFC emulsions (PEs), EGF-loaded nanoparticles (ENPs), and the antimicrobials polyhexamethylene biguanide (PHMB) named P_E_E_NP_PCH for diabetic wound treatment, in which individual encapsulation of EGF and PFC may prevent their interactions with PHMB and/or with each other before they are released to the wound site. Furthermore, P_E_E_NP_PCH may exert additional anti-inflammatory effects due to the anti-inflammatory characteristics of chitosan reported previously [24]. With capabilities of oxygen delivery, antimicrobial activity, anti-inflammation, and promotion of cell growth, we anticipate that the developed P_E_E_NP_PCH may be able to provide improved efficacy in diabetic wound repair. In this paper, the manufacture of P_E_E_NP_PCH was first introduced, followed by sequential investigations of its physicochemical properties, functions, and therapeutic effectiveness in diabetic wound healing in vitro and in vivo.

## 2. Materials and Methods

### 2.1. Preparation and Evaluation of PEs and ENPs

PEs were fabricated by homogenizing perfluorooctyl bromide (PFOB; Sigma, Saint Louis, MO, USA) and Pluronic F-68 (Sigma, Saint Louis, MO, USA) solution (10% (*w/v*)) in a volume ratio of 9:1 (W/PFC) in an ice bath for 10 min. In this study, the number of PEs was estimated using gravimetric measurements and the regression analysis reported previously [25].

**Figure 1:**
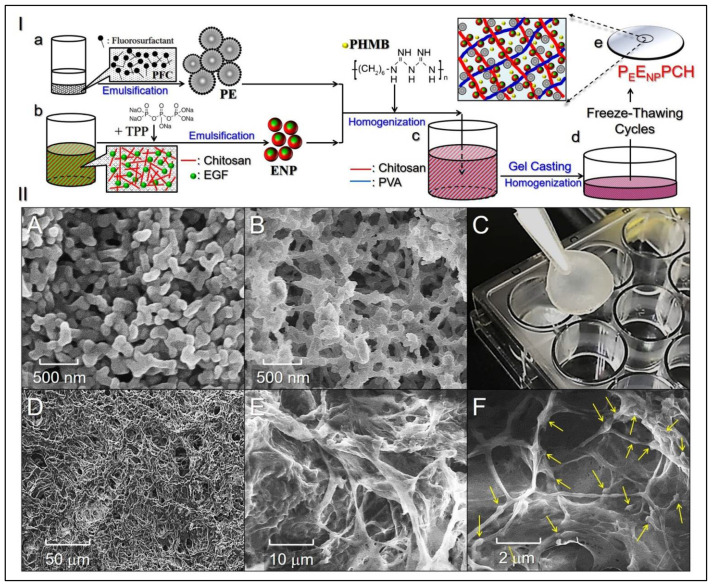
Analyses of the structure and morphology of the P_E_E_NP_PCH. (**I**) Schematic diagram of the P_E_E_NP_PCH fabrication procedures, including the manufacture of PEs and ENPs (**a**,**b**), hetero-composite hydrogel formulation (**c**), casting (**d**), and formation after eight freeze–thaw cycles (**e**). (**II**) Photomicrographic images of the manufactured nanoparticles and hetero-composite hydrogel. (**A**,**B**) SEM images of the ENPs (**A**) and PEs (**B**) at 50,000× magnification. (**C**) Photograph of the real P_E_E_NP_PCH sample. (**D**–**F**) SEM images showing the surface of the P_E_E_NP_PCH at 500× magnification (**D**), the inner structure of the P_E_E_NP_PCH at 2000× magnification (**E**), and the inner structure of the P_E_E_NP_PCH at 10,000× magnification (**F**). Arrows indicate the nanoparticles (ENPs and PEs) adhered to the polymeric fibers.

A total of 100 μg of human EGF were first mixed with chitosan (50–160 kDa) in acetic acid (3% (*w/v*)), and then the mixture was added dropwise to sodium tripolyphosphate (TPP, 0.1% (*w/v*)) under magnetic agitation to fabricate ENPs. After stirring with 2000 rpm at ambient temperature for 4 h, the obtained ENPs were washed by centrifugation and resuspended in deionized (DI) water. The procedures used to fabricate the PEs and ENPs are illustrated in Figure 1(a,b).

The size distribution and surface charge of the PEs and ENPs were separately measured using dynamic light scattering (DLS, NanoBrook 90plus, Brookhaven Instruments, Holtsville, NY, USA). The morphologies of the PEs and CNP_E_ were detected using scanning electron microscopy (SEM, HITACHI SU8200, Hitachi Ltd., Tokyo, Japan). The encapsulation efficiency (*E*_E_; %) and the loading ratio (*L*_E_; %) of the EGF in the ENPs were calculated using the following equations [26]:

(1) $$E_{E}=\frac{W-W_{u}}{W}\times 100\%$$

(2) $$L_{E}=\frac{W_{E}}{W_{P}}\times 100\%$$

where *W* is the total amount of EGF used for ENP manufacturing. *W*_u_ denotes the amount of unencapsulated molecules determined using the human EGF ELISA kit (RayBiotech, Peachtree Corners, GA, USA). *W*_E_ denote the weight of the entrapped EGF in the ENPs, and *W*_P_ is the weight of the ENPs.

### 2.2. Fabrication of the P_E_E_NP_PCH

The P_E_E_NP_PCH was fabricated using a modified freeze–thaw cycling method reported previously [27]. Briefly, 1 mL of DI water containing PHMB, PEs, and ENPs with designated doses of PHMB, PFOB, and EGF was first mixed with chitosan (50–160 kDa, 3% *w/v* in 0.5 M acetic acid) and polyvinyl acetate (PVA; 10 wt% in DI water) solutions in a 1/2/3 (*v/v*) ratio of chitosan/heterogeneous mixture/PVA. After complete dissolution, the mixed solution was poured into a casting mold and subjected to eight freeze–thaw cycles to form the P_E_E_NP_PCH. The first run of the freeze–thaw process was operated by incubating the sample at 25 °C for 30 min and −20 °C for 12 h, while the following 7 cycles were all performed by incubating the sample at 25 °C for 30 min and −20 °C for 1 h. The surface and inner structures of the P_E_E_NP_PCH were detected using SEM. Figure 1(a–e) shows all the procedures used to manufacture the P_E_E_NP_PCH.

### 2.3. Evaluation of the Mechanical and Thermal Properties of the P_E_E_NP_PCH

The stress–strain capability of the hydrogel sample with a size of 4.5 × 1.7 × 0.2 cm (L/W/H) was measured using an Instron 4467 testing machine (Instron, Norwood, MA, USA). The maximal load of the sample was evaluated by setting a 6 mm/min extension rate with a 10 N preload. The thermal properties of P_E_E_NP_PCH were assessed using thermogravimetric analysis (TGA) (PYRIS 1, Perkin Elmer, Waltham, MA, USA) in association with derivative thermogravimetry (DTG), where the lyophilized sample was heated from 50 to 800 °C at a rate of 10 °C/min under continuous nitrogen flow.

### 2.4. Assessment of the Hydration Capability of the P_E_E_NP_PCH

Both the swelling degree (SD) and equilibrium water content (EWC) of the P_E_E_NP_PCH were assessed using the gravimetrical approach. Briefly, the hetero-composite hydrogel was first dehydrated and weighed immediately after lyophilization. The dried sample was then immersed in PBS (pH = 7.4) at 37 °C and weighed at 2, 4, 8, 12, 24, and 48 h after gently removing the excess water on the sample surface. The SD and EWC of the hydrogel at each time point were calculated using the following equations [28]:

(3) $$\mathrm{SD}=\frac{W_{t}-W_{0}}{W_{0}}\times 100\%$$

(4) $$\mathrm{EWC}\;=\frac{W_{t}-W_{0}}{W_{t}}\times 100\%$$

where *W*_0_ represents the dry weight of the hydrogel at *t* = 0. *W_t_* denotes the weight of the swelled hydrogel after water absorption at a specific time *t* > 0.

### 2.5. Analysis of In Vitro Degradation of the P_E_E_NP_PCH

The in vitro degradations of blank chitosan hydrogel (CH) and P_E_E_NP_PCH were evaluated by measuring the weight loss of the samples over time as reported previously [29]. For each type of hydrogel, 8 pre-weighed samples were separately immersed in PBS and maintained in a 37 °C orbital shaking incubator (OSI-500, DEAGLE company, New Taipei, Taiwan) set at 50 rpm. After 0, 2, 4, 6, 12, and 24 h followed by every 24 h to the 7th day, the PBS was removed, and the samples were dried until a constant weight was obtained. The percentage of weight loss (*L_W_*) of each sample was calculated using the equation [29]:

(5) $$L_{W}\;=\frac{W_{I}-W_{dt}}{W_{I}}\times 100\%$$

where *W_I_* is the initial weight of the sample and *W_dt_* is the weight of the sample after drying at a specific time *t* > 0.

### 2.6. Analyses of the Kinetics of PHMB and EGF Release from the P_E_E_NP_PCH

P_E_E_NP_PCHs containing 25 mg/mL PFOB, 2000 ppm PHMB, and 60 µg/mL EGF were immersed in PBS and DI water separately at 37 °C. After 0, 2, 4, 8, 12, 24, and 48 h, the amounts of PHMB and EGF released in the supernatants were analyzed using a UV–VIS spectrometer (V-650, JASCO, Easton, MD, USA) set at λ_abs_ = 235 nm and the human EGF ELISA kit (RayBiotech, Peachtree Corners, GA, USA), respectively.

### 2.7. Bacterial Cultivation

Both *Staphylococcus aureus* (*S. aureus*; ATCC^®^ 23235^TM^) and *Staphylococcus epidermidis* (*S. epidermidis*; ATCC^®^ 12228^TM^) were cultivated in tryptic soy broth (TSB) under aerobic conditions at 37 °C. The quantity of each type of bacteria was determined using spectrophotometry at λ_abs_ = 600 nm. Bacteria were diluted 1:100, and sub-cultivation was performed as the value of the absorbance (optical density; OD_600_) was ≥1.0.

### 2.8. Cell Culture

Human keratinocytes (KERTr cells; ATCC^®^ CRL-2309™) were cultured with keratinocyte serum-free medium (KSFM) supplemented with bovine pituitary extract and human recombinant epidermal growth factor at 37 °C with 5% CO_2_ and 100% humidity.

### 2.9. Assessment of the Oxygen Delivery Capacity of the P_E_E_NP_PCH

The oxygen delivery capacity of the hydrogel was detected using resazurin as the oxygen indicator [30]. A transparent/anaerobic resazurin-sodium sulfide medium (RSSM) was first prepared by adding 40 μL of resazurin (4.4 mM) to 40 mL of a sodium sulfide solution (1.2 M), followed by heating the mixture at 50 °C for 20 min. Afterward, 0.5 cm^3^ of the P_E_E_NP_PCH containing 25 mg/mL PFOB, 2000 ppm PHMB, and 60 µg/mL EGF was placed in 10 mL of RSSM at ambient temperature. Next, 0.5 mL of DI water, PEs, and E_NP_PCH (obtained using the same fabrication method as the P_E_E_NP_PCH without adding PEs) with equal doses of PFOB, PHMB, and EGF (if present) were employed for comparison. The color variations of the solutions were monitored and photographed every minute for 10 min.

### 2.10. Assessment of the Antibacterial Effect and Cytotoxicity of the P_E_E_NP_PCH In Vitro

The antibacterial capability of the P_E_E_NP_PCH was evaluated using both inhibition zone assay and colony assay [31,32]. In the former approach, P_E_E_NP_PCHs containing 0, 500, 1000, 2000, and 4000 ppm PHMB were separately placed in the center of the bacterial agar plate on which *S. aureus* or *S. epidermidis* were spread in 50 µL of TSB with OD_600_ = 0.3. After an incubation at 37 °C for 24 h, the clear area around the hydrogels was quantitatively measured using ImageJ software. For the colony assay, bacteria (*S. aureus* or *S. epidermidis*) with an OD_600_ = 0.3 were first co-cultured with P_E_E_NP_PCHs containing 0, 500, 1000, 2000, or 4000 ppm PHMB for 12 h, and then different dilutions of bacteria were separately dropped on TSB agar plates. After culture at 37 °C for 12 h, the antimicrobial capability of each group was determined based on the value of the microbial population index (MPI) defined by the logarithm of the colony formation unit (CFU; MPI = Log_10_ ((CFU + 1)/mL)) [32].

P_E_E_NP_PCHs containing 0, 500, 1000, 2000, and 4000 ppm PHMB were separately immersed in cell culture medium at 37 °C for 48 h, and then the supernatants were collected, centrifuged, and used for KERTr cell culture to evaluate the cytotoxicity of the heterogeneous hydrogel. After treatment for 24 h, cell viability was analyzed using a hemocytometer with the trypan blue exclusion method. In this cytotoxicity study, the doses of PFOB and EGF in the P_E_E_NP_PCHs were set to 25 mg/mL and 60 µg/mL, respectively.

### 2.11. Evaluation of the Anti-Inflammatory Effect of the P_E_E_NP_PCH In Vitro

Two types of conditional media were prepared prior to the experiment. First, inflammation-induced medium (IIM) was prepared by mixing cell culture medium and TSB, which was used for *S. aureus* cultivation for 24 h and purified by centrifugation and filtration, at 1:200 (*v/v*). P_E_E_NP_PCH containing 25 mg/mL PFOB, 60 µg/mL EGF, and 2000 ppm PHMB was immersed in the IIM at 37 °C for 48 h, and then the supernatant (i.e., P_E_E_NP_PCH-treated IIM; P-IIM) was collected, centrifuged, and stored at 4 °C until use. Afterward, the KERTr cells were separately treated with normal media (control), IIM, P-IIM, or IIM + N-acetyl-D-glucosamine (NADGA; 10 mM) at 37 °C for 12 h and then cellular interleukin-8 (IL-8) expression was analyzed using the human IL-8/CXCL8 ELISA kit (R&D System, Inc., Minneapolis, MN, USA).

### 2.12. Evaluation of Effect of the P_E_E_NP_PCH on Cell Growth

P_E_E_NP_PCHs containing 0.6, 6, and 60 μg/mL EGF were separately immersed in KSFM at 37 °C for 48 h, and then the supernatants (P_E_E_NP_PCH-treated KSFMs; P-KSFMs) were collected, centrifuged, and stored at 4 °C until use. Afterward, photomicrographs of the KERTr cells treated with KSFM (control) or different P-KSFMs were captured and the cell number was counted every 24 h for 7 days. The cell growth rate (*μ*) of each group was calculated using the equation [33]:

(6) $$\mu \times (t_{2}-t_{1})=\ln\left(\frac{C_{t2}}{C_{t1}}\right)$$

where *C*_*t*1_ and *C*_*t*2_ denote the cell concentrations detected at time points *t*_1_ and *t*_2_, respectively.

### 2.13. Animal Study

A total of 30 Sprague–Dawley rats (8–10 weeks) weighing between 250 and 300 g were used for the in vivo diabetic wound healing study. All animal procedures, including the care and operation of laboratory animals, were performed in accordance with the guidelines approved by the Cathay General Hospital (Taiwan, Approval number: IACUC 110-001. Approval Date: 28 December 2020). Diabetes mellitus was induced in rats by administering a single intravenous injection of 65 mg/kg streptozotocin (STZ), and rats with blood glucose levels > 300 mg/dL on the 21st day were considered diabetic [34]. After anaesthetization via isoflurane inhalation, the dorsal skin of each rat was shaved and sanitized, and then two identical circular full-thickness wounds with d = 0.8 cm were created using a punch. One of the two wounds on each rat was covered with sterilized gauze, while the other wound was treated with the commercial dressing HeraDerm (Amed, New Taipei City, Taiwan), blank CH, E_NP_PCH, P_E_PCH, or P_E_E_NP_PCH. Dressings were changed every 72 h throughout the experiment. The conditions of the wounds were photographed before changing the new dressings. The degree of wound closure (DWC) of each group was estimated using the equation [35]:

(7) $$\mathrm{DWC}=\left(\frac{A-A_{t}}{A}\right)100\%$$

where *A* is the area of the wound at Day 0. *A_t_* denotes the wound area at a specific time *t* > 0 obtained using ImageJ software. The experimental rats were sacrificed on the 15th day.

### 2.14. Histological Study

Dorsal skin sample around the wound were harvested by dissection with sharp scissors immediately after the rats were sacrificed. All tissue specimens were sequentially fixed with formalin, dehydrated, cleared with xylene, infiltrated with wax, and embedded in paraffin. A 5-μm section of each paraffin block was separately stained with hematoxylin and eosin (H&E), interleukin-1 beta (IL-1β) immunohistochemistry (IHC), and Masson’s trichrome, followed by image analysis using Motic DSA software (Motic, Kowloon, Hong Kong). Furthermore, the alignment and orientation of the regenerating collagen fibers observed in each Masson’s trichrome-stained image were analyzed and quantitatively measured using small angle light scattering (SALS, TA Instruments, New Castle, DE, USA) in association with anisotropy index (AI) approach reported previously [36].

### 2.15. Statistical Analysis

All data were acquired from ≥three independent experiments and were presented as the mean ± standard deviation (s.d.). Statistical analyses were conducted using MedCalc software in which comparisons of 1 condition between 2 groups were performed using Student’s *t*-test followed by Dunnett’s post-hoc test at a significance level of *p* < 0.05 throughout the study.

## 3. Results and Discussion

### 3.1. Characterization of the PEs, ENPs, and P_E_E_NP_PCH

Figure 1II(A) and Figure 1II(B) show SEM images of ENPs and PEs, respectively, and both nanocarriers retained an intact particulate morphology after the rigorous fabrication process. The size and surface charge of the ENPs were 251 ± 40.5 nm and 28.6 ± 2.23 mV, whereas those of the PEs were 160 ± 13.5 nm and −0.23 ± 1.01 mV, respectively. The encapsulation efficiency and loading ratio of EGF in the ENPs were 86.9 ± 1.01% and 0.087 ± 0.013 wt%, respectively.

The P_E_E_NP_PCH is a moist and semitransparent membrane (Figure 1II(C)) with a porous morphology on both surface and internal structures, as illustrated in Figure 1II(D,E). Moreover, it can be seen that a number of nanoparticles (ENPs and PEs) were adhered to the fibrous surface and/or entrapped inside the fibers throughout the hydrogel matrix (Figure 1II(F)), illustrating the heterogeneous construction of the P_E_E_NP_PCH.

### 3.2. Mechanical Property, Thermal Property, and Degradation of the P_E_E_NP_PCH

**Figure 2:**
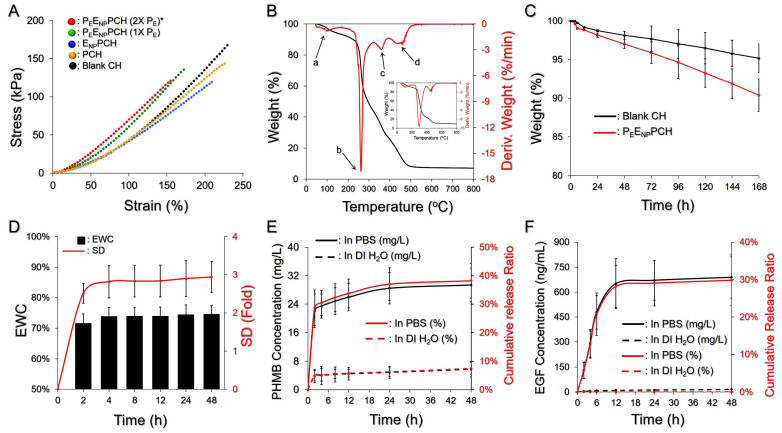
Assessment of the physicochemical properties of P_E_E_NP_PCH. (**A**) Stress vs. strain curves of the four hydrogels indicated in the figure. The doses of EGF and PHMB in the three hetero-composite hydrogels were equally set to 60 μg/mL and 2000 ppm, respectively. * 2X PEs denotes that the dose of PE-trapped PFOB in the hydrogel was 50 mg/mL. (**B**) TGA and DTG curves of the P_E_E_NP_PCH at temperatures ranging from 50–800 °C were obtained at a heating rate of 10 °C/min under a nitrogen atmosphere. The inset image represents the TGA and DTG curves of the blank CH. (**C**) Degradation profiles of the P_E_E_NP_PCH (red) and the blank CH (black) in 37 °C PBS within 7 days. (**D**) The swelling profile (red) and the EWC (black) of the P_E_E_NP_PCH in 37 °C PBS within 48 h. (**E**,**F**) In vitro kinetic drug release profiles of PHMB (**E**) and EGF (**F**) from the P_E_E_NP_PCH incubated in PBS or DI water at 37 °C within 48 h. The cumulative released amount and ratio for each drug are presented in black and red curves, respectively, as indicated in the figure. Values in (**C**–**F**) are presented as mean ± s.d. (n = 3).

Figure 2A presents the mechanical properties of P_E_E_NP_PCH containing various concentrations of PHMB, ENPs, and/or PEs. Compared to the blank CH, our data show that incorporation of PEs/ENPs increased the stiffness of the hydrogel and that both tensile strength (stress) and elasticity (strain) were reduced with an increase of the number of nanoparticles loaded. A likely explanation for this finding is that the presence of nanoparticles may hinder the formation of fibers and/or cause collapse of fibrous networks since they were tightly incorporated with the fibers as shown in Figure 1II(F).

Figure 2B shows the TGA/DTG curves of the developed hydrogels at temperatures ranging from 50 to 800 °C. In contrast to the blank CH (Figure 2B, inset), four-stage thermal degradation was observed for the P_E_E_NP_PCH as indicated by the four weight loss peaks in the corresponding DTG profile. The first stage occurred between 80 and 200 °C with ~15% weight loss and was likely attributed to the losses of adsorbed water, acetic acid, PHMB (*T*_m_~91 °C; decomposition temperature~190 °C), and PFOB (*T*_b_~142 °C) [37,38]. The second stage occurred between 210 and 280 °C with 45% weight loss and was strongly correlated with the crystalline fraction of PVA and deacetylation level of the chitosan utilized for ENP and P_E_E_NP_PCH manufacturing, as reported previously [39]. The third weight loss of ~20% at 310–380 °C was reasonably attributed to further degradation of chitosan and decomposition of Pluronic F68 [40]. The fourth weight loss of ~10% at 400–600 °C likely resulted from further degradation of the remaining Pluronic F68 and TPP (*T*_m_~620 °C).

Figure 2C shows in vitro degradation profiles of the blank CH and the P_E_E_NP_PCH under 37 °C PBS within seven days. The *L_W_* of the blank CH was 4.83 ± 1.9%, while that of the P_E_E_NP_PCH was 9.63 ± 2.6% after seven days. We speculated that the increase of weight loss in the P_E_E_NP_PCH was due to addition of functional components including PHMB, PEs, and ENPs. Since chitosan has been known to be able to interfere crosslinking of PVA during freeze-thaw cycles [41], interactions between PVA and ENPs may further hinder PVA crosslinking and thereby reduce the strength of the P_E_E_NP_PCH network, leading to an increased weight loss compared to the blank CH.

### 3.3. Hydration and Drug Release Kinetics of the P_E_E_NP_PCH

Figure 2D shows the hydration capacity of the dehydrated P_E_E_NP_PCH in PBS (pH = 7.4) at 37 °C. The P_E_E_NP_PCH quickly swelled within the first 2 h and was maintained at a similar level beginning at 4 h, showing an approximately 3-fold SD and a 75% EWC after 48 h. Based on these results, the P_E_E_NP_PCH effectively absorbed ion-rich medium to >70% of the total weight, showing that the developed hetero-composite hydrogel is highly applicable for use to take up body fluids/exudates and to maintain a moist environment around the wound site.

Figure 2E,F show the kinetic profiles of PHMB and EGF release from the P_E_E_NP_PCH in PBS or DI water within 48 h. The hydrogel in PBS exhibited a two-phase release profile for both agents, obtaining 29.4 mg/L (Figure 2E) and 688.3 ng/mL (Figure 2F) cumulative released concentrations after 48 h, corresponding to 40% and 30% cumulative release percentages for PHMB and EGF, respectively. However, the drug release from the P_E_E_NP_PCH in DI water was highly restricted, and the amounts of PHMB and EGF released were all <10% within 48 h. These outcomes clearly show that the P_E_E_NP_PCH is susceptible to the ionic strength of the environment. We reason that this finding was attributed to the increased ionic interactions between electrolytes, amine groups of chitosan, and acetic groups of PVA that may reduce the chitosan-PVA crosslinking affinity and thus increase the efficiency of water transportation in and out of the hydrogel, consequently leading to accessible drug delivery [42].

### 3.4. Oxygen Delivery by the P_E_E_NP_PCH

**Figure 3:**
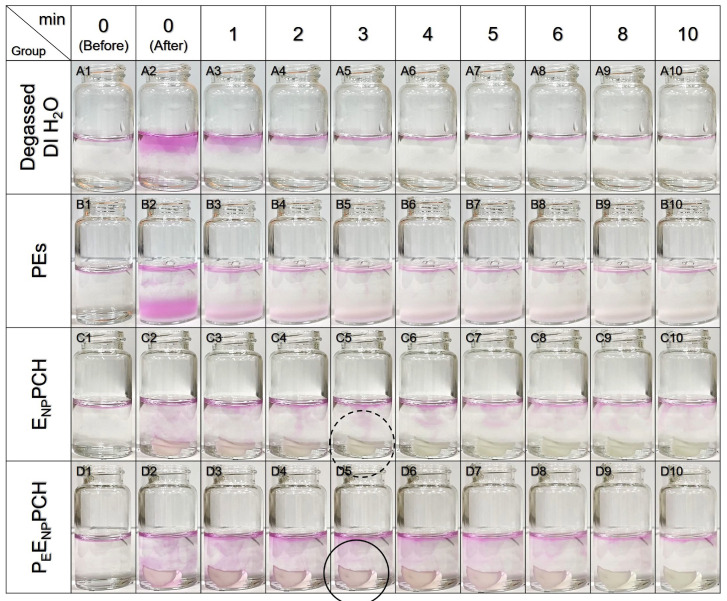
Assessment of the O_2_-carrying capacity of the P_E_E_NP_PCH. Images of 10 mL of RSSM before and after adding 100 μL of degassed DI water (Row A), 100 μL of PEs (Row B), E_NP_PCH (*V* = 0.5 cm^3^; Row C), or P_E_E_NP_PCH (*V* = 0.5 cm^3^; Row D) for 10 min. The doses of PFOB in the PEs (25 mg/mL); or EGF (60 μg/mL) and PHMB (2000 ppm) in the E_NP_PCH, were all equal to those in the P_E_E_NP_PCH. The pink color denotes the presence of oxygen in the region. The E_NP_PCH was colorless while the P_E_E_NP_PCH retained pink at the 3rd min as indicated by dashed and solid circles, respectively.

The oxygen delivery capability of the P_E_E_NP_PCH was qualitatively detected using resazurin, with a pink color serving as the indicator, and the results are shown in Figure 3. The pink color observed at every liquid–gas interface was attributed to the dissolution of atmospheric oxygen rather than immersion of the sample material as illustrated in Figure 3(A1–D1). Compared to the group incubated with degassed DI water, which showed a transparent liquid throughout the time course (Figure 3(A2–A10)), the group treated with PEs ([PFOB] = 25 mg/mL) exhibited pink color for 10 min (Figure 3(B2–B10)) and that was surely attributed to the constituent PFC. Similarly, the P_E_E_NP_PCH with 25 mg/mL PFOB sustainably appeared pink for 10 min (Figure 3(D2–D10)), while the pink color generated by E_NP_PCH only lasted for 2 min (Figure 3(C1–C5)). These results clearly showed that the P_E_E_NP_PCH containing ≥25 mg/mL PFOB may deliver oxygen that is helpful for reducing hypoxia in diabetic wounds. Regarding the question of why E_NP_PCH without PFC exhibited an oxygenated response in the first 2 min, we reason that this finding was attributed to the entrapment of a small amount of oxygen in the hydrogel during fabrication that conferred a short oxygen response to E_NP_PCH.

### 3.5. Antibacterial and Cytotoxic Effects of the P_E_E_NP_PCH In Vitro

**Figure 4:**
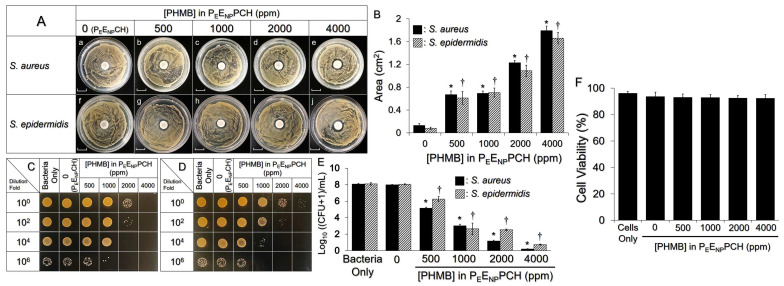
Antimicrobial and cytotoxic effects of P_E_E_NP_PCH containing various doses of PHMB. (**A**) Images of the inhibition zone of *S. aureus* (**a**–**e**) or *S. epidermidis* (**f**–**j**) generated by the P_E_E_NP_PCH containing 0 (P_E_E_NP_CH), 500, 1000, 2000, and 4000 ppm PHMB. Each group was photographed after treatment with the hydrogel for 24 h. Scale bar = 1.0 cm. (**B**) Quantitative analyses of the inhibition zones shown in (**A**). (**C**,**D**) Images of *S. aureus* (**C**) and *S. epidermidis* (**D**) colonies after treatment with P_E_E_NP_CH or P_E_E_NP_PCH containing various PHMB dosages as indicated in the figure. Images in the four rows represent the colony forming conditions of the group using bacteria with different dilutions as the seed. All images were captured 12 h after inoculation on TSB agar plates. (**E**) Quantitative analyses of the MPI of *S. aureus* or *S. epidermidis* shown in (**C**,**D**). Values are presented as mean ± s.d. (n = 3). * *p* < 0.05 compared to the *S. aureus* group treated with the P_E_E_NP_CH. ^†^ *p* < 0.05 compared to the *S. epidermidis* group treated with the P_E_E_NP_CH. (**F**) Toxicity of P_E_E_NP_PCH containing 0 (i.e., P_E_E_NP_CH), 500, 1000, 2000, or 4000 ppm PHMB to KERTr cells after treatment for 24 h. Values are presented as the mean ± s.d. (n = 3).

Figure 4A,B show the antibacterial effects of the P_E_E_NP_PCH containing various doses of PHMB investigated using the inhibition zone assay. For both bacterial strains, the groups treated with the P_E_E_NP_PCH containing ≥500 ppm PHMB generated a noticeable clean region around the hydrogel compared to the hydrogel without PHMB (i.e., P_E_E_NP_CH; Figure 4A), and the inhibition area was increased by ~3-fold as the concentration of PHMB was increased from 500 to 4000 ppm (Figure 4B). Similar results were obtained in the colony assay (Figure 4C,D). Compared to the P_E_E_NP_CH-treated group, the MPI of the P_E_E_NP_PCH-treated groups of both *S. aureus* and *S. epidermidis* was significantly decreased when the PHMB dosage was ≥500 ppm (*p* < 0.05; Figure 4E), and a >90% reduction in the MPI was obtained when the dose of PHMB was increased to 4000 ppm (Figure 4E).

The cytotoxicity of the P_E_E_NP_PCH containing different PHMB doses toward KERTr cells was further examined using a noncontact approach. As plotted in Figure 4F, all groups showed >90% viability, indicating that the in vitro cytotoxicity of the P_E_E_NP_PCH containing ≤4000 ppm PHMB was negligible. Taken together, these outcomes suggest that the P_E_E_NP_PCH containing 500–4000 ppm PHMB may provide robust antimicrobial capability without noticeable in vitro cytotoxicity. For an effective antimicrobial activity without unpredicted toxicity when the P_E_E_NP_PCH was utilized in vivo, 2000 ppm PHMB was selected as the antibiotic dosage of the P_E_E_NP_PCH in subsequent studies.

### 3.6. Effect of the P_E_E_NP_PCH on Promoting Cell Growth

**Figure 5:**
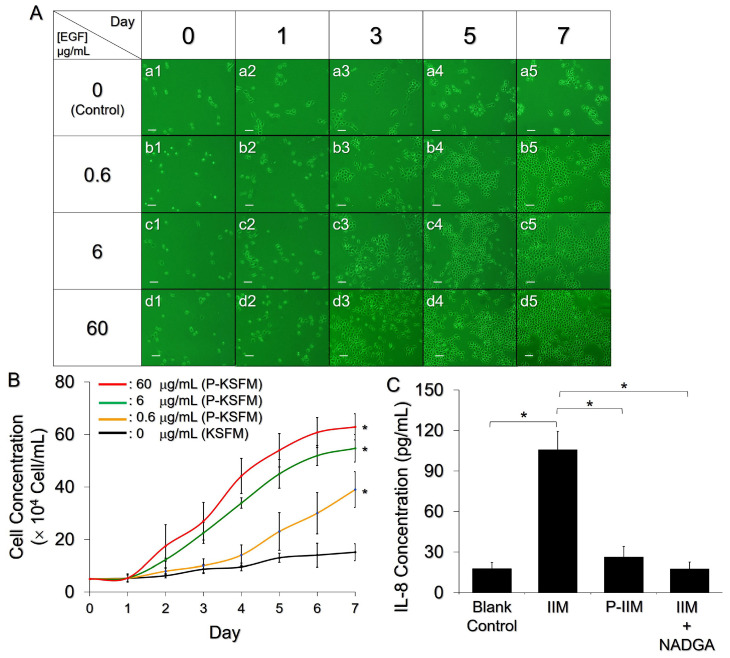
Effects of the P_E_E_NP_PCH on cell growth and the inflammatory response. (**A**) Photomicrographs of KERTr cells after treatment with P-KSFM for 0, 1, 3, 5, and 7 days, in which the P_E_E_NP_PCHs were prepared with 0.6, 6, or 60 μg/mL EGF. The group cultured with KSFM alone ([EGF] = 0 μg/mL) was employed as the control. Scale bar = 50 μm. (**B**) The kinetic profiles of KERTr cell growth after treatment with KSFM or various P-KSFMs within seven days. Values are presented as mean ± s.d. (n = 3). * *p* < 0.05 compared to the group with KSFM alone. (**C**) IL-8 expression levels in the KERTr cells after treatment with control media, IIM, P-IIM, or IIM + NADGA for 12 h. Values are presented as the mean ± s.d. (n = 3). * *p* < 0.05.

With a determined dose of PHMB, the effect of entrapped EGF on cell proliferation was subsequently examined using P-KSFM as the growth stimulus. As shown in Figure 5A, KERTr cells exhibited EGF dose-dependent cell growth within 7 days. Compared to the group without EGF (Figure 5A(a1–a5), *μ* = 0.175 day^−1^, Figure 5B), the KERTr cells treated with the P-KSFM prepared using the P_E_E_NP_PCH containing 0.6, 6, and 60 μg/mL EGF exhibited 1.9-, 2.2-, and 2.4-fold enhancement in cell growth rate within seven days, resulting in 2.6-, 3.6-, and 4.2-fold (*p* < 0.05 for each, Figure 5B) increases in cell numbers on the 7th day, respectively. Based on these results, the P_E_E_NP_PCH containing ≥0.6 µg/mL nanoparticle-encapsulated EGF was indeed able to facilitate keratinocyte proliferation, which is foreseeably favorable for tissue regeneration at the wound site. We ensured increased cell growth in vivo by adding 60 µg/mL of EGF in the ENPs in subsequent P_E_E_NP_PCH studies.

### 3.7. Anti-Inflammatory Effect of the P_E_E_NP_PCH

The anti-inflammatory effect of the P_E_E_NP_PCH containing the indicated doses of PFOB, EGF, and PHMB was further examined by measuring IL-8 expression in the stimulated KERTr cells. As shown in Figure 5C, the IL-8 expression level in the group treated with P-IIM was similar to that of the IIM+NADGA-treated group (*p* = NS) and was 4-fold (*p* < 0.05) lower than that of the IIM-treated group, showing that the P_E_E_NP_PCH was able to reduce the inflammatory response caused by *S. aureus*. We reason that the anti-inflammatory effect of P-IIM was mainly attributed to the chitosan molecules released from the P_E_E_NP_PCH. Since free chitosan is very likely to be depolymerized during P-IIM preparation at 37 °C (e.g., acid hydrolysis) [43], hydrolyzed/degraded chitosan with a low MW (e.g., chitosan oligosaccharide) will likely be present in the P-IIM and confer anti-inflammatory activity, as reported previously [44,45].

### 3.8. Diabetic Wound Healing Effect of the P_E_E_NP_PCH In Vivo

**Figure 6:**
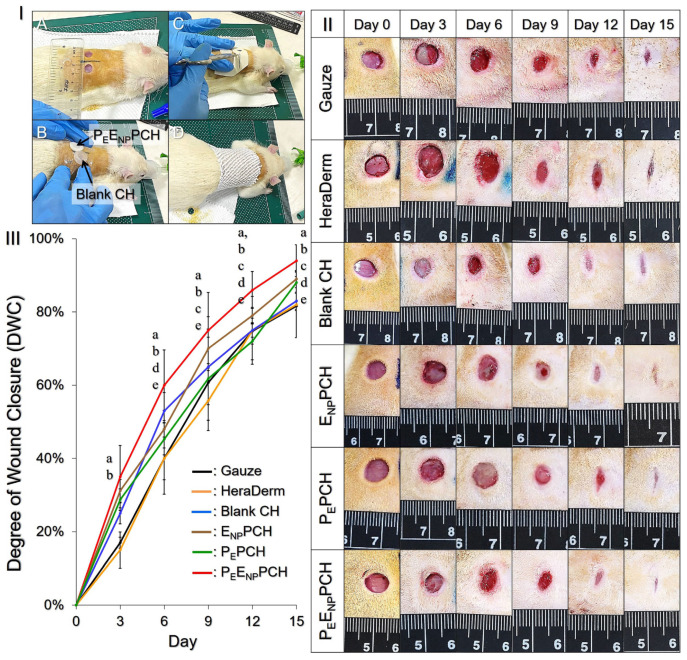
Wound healing efficacy of the P_E_E_NP_PCH in vivo. (**I**) Photographs showing the animal operations including wound generation (**A**), coverage with the dressing (**B**), fixation of the dressing (**C**), and wound bandaging (**D**). (**II**) Images of the wound conditions of the diabetic rats receiving various treatments for 15 days. Wounds were photographed every 72 h for 15 days before replacing the new dressings. (**III**) Quantitative analysis of the DWC of each group within 15 days. Values are presented as mean ± s.d. (n = 5). a, b, c, d, and e represent *p* < 0.05 compared to the groups treated with gauze, HeraDerm, blank CH, E_NP_PCH, and P_E_PCH, respectively.

With verified in vitro functionalities, including antibacterial activity, anti-inflammation, oxygen delivery, and promotive cell growth, the effect of the P_E_E_NP_PCH on wound healing in vivo was further explored using diabetic rats (Figure 6I). As presented in Figure 6II, the wound treated with P_E_E_NP_PCH quickly healed and displayed the smallest visible size among all groups after 15 days. Based on the quantitative analysis of the wound area (Figure 6III), the wound treated with P_E_E_NP_PCH showed the greatest DWC of 35% while that of all the others remained <30% at Day 3, and enabled a 95% DWC at Day 15 that was 15.2%, 14.6%, 13.3%, 5.6%, and 6.8% (*p* < 0.05 for all) higher than the values gained from the groups treated with gauze, HeraDerm, blank CH, E_NP_PCH, and P_E_PCH, respectively. These results clearly demonstrated that the P_E_E_NP_PCH was truly effective in healing wounds on diabetic rats and all the functional components, including PEs/PFOB, ENPs/EGF, and PHMB were essential for enhanced wound recovery. Furthermore, unlike gauze and HeraDerm, which would adhere to the wound tissues, the P_E_E_NP_PCH was less adhesive due to its moist character and thus was able to reduce pain and avoid secondary damage to the regenerating tissues during dressing replacement that is highly advantageous for wound care.

### 3.9. Histological Analysis

**Figure 7:**
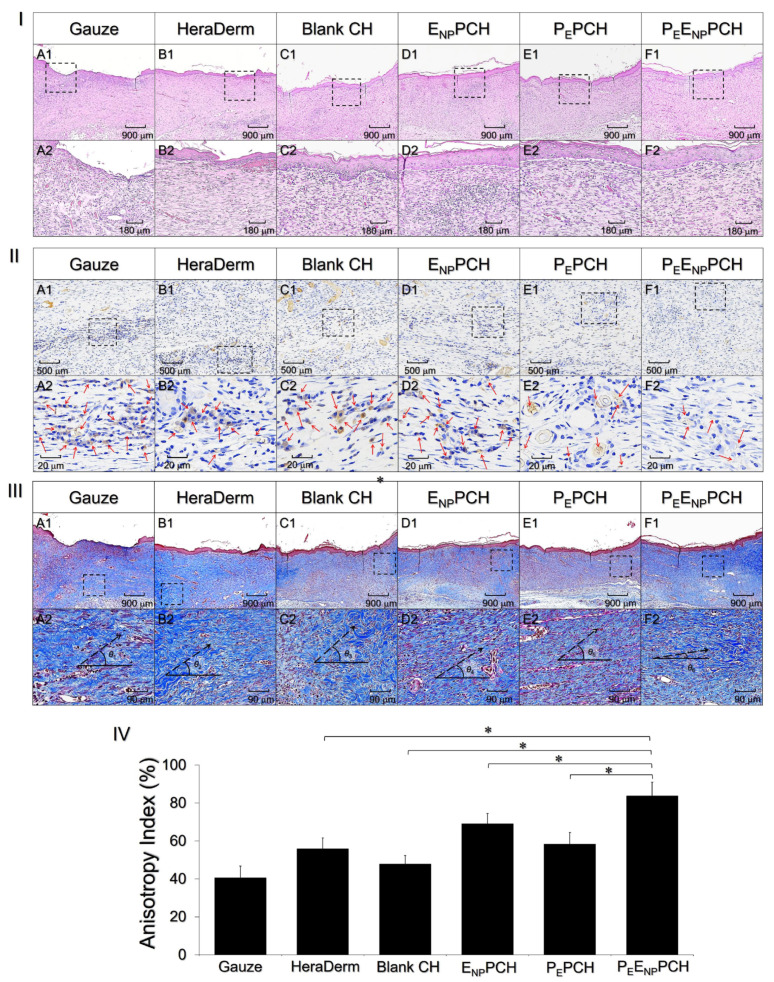
Histological analyses of the skin tissues recovered from the wounds. (**I**–**III**) Photomicrographs of regenerated tissues stained with H&E (**I**), IL-1β IHC (**II**), and Masson’s trichrome (**III**) after administration of different treatments for 15 days as indicated in the figure. Images (**A1**–**F1**) in (**I–III**) were photographed at 40× magnification. Photographs (**A2**–**F2**) in (**I**–**III**) are higher magnification images of the areas indicated by the dashed blocks shown in (**A1**–**F1**), respectively. Red arrows in (**A2**–**F2**) in (**II**) denote the locations with IL-1β expression shown in brown color. *θ*_1_–*θ*_6_ and dashed arrows in (**A2**–**F2**) in (**III**) denote the collagen alignment angles and the orientations of the collagen fiber alignment, respectively. (**IV**) AI analyses of the collagen fiber alignment shown in (**A2**–**F2**) in (**III**). Values are presented as mean ± s.d. (n = 5). * *p* < 0.05.

The conditions of the newly formed tissues beneath the wounds were further investigated by performing a histological analysis. As shown in Figure 7I, the groups treated with the E_NP_PCH (Figure 7ID) and P_E_E_NP_PCH (Figure 7IF) exhibited a relatively complete/intact epithelial layer and fewer wrinkles than those treated with gauze (Figure 7IA), HeraDerm (Figure 7IB), blank CH (Figure 7IC), and P_E_PCH (Figure 7IE), while the groups treated with the P_E_PCH (Figure 7IIE) and P_E_E_NP_PCH (Figure 7IIF) showed a relatively mild inflammatory response compared to all the other treatments (Figure 7IIA–D). Wounds in all groups were re-epithelialized and exhibited collagen deposition after 15 days, according to Masson’s trichrome staining (Figure 7IIIA–F). However, the degree of wound recovery was considered by measuring not only the amount of collagen produced but also its maturation level, which is strongly related to the functionality and integrity of the regenerated tissues [46].

According to the micrographic detection, most of the regenerating collagen fibers in the group treated with the P_E_E_NP_PCH were aligned in a more horizontal manner and showed a smaller alignment angle (*θ*_6_; Figure 7IIIF2) compared to that in the other five groups (*θ*_1_–*θ*_5_, Figure 7IIIA2–E2). Based on the SALS analyses plotted in Figure 7IV, the group treated with the P_E_E_NP_PCH exhibited the highest AI of 83.7 ± 7.3%, which was 2.1-, 1.5-, 1.8-, 1.2-, and 1.4-fold (*p* < 0.05 for each) higher than the values obtained using gauze, HeraDerm, blank CH, E_NP_PCH, and P_E_PCH, respectively.

These results imply that ENPs and PEs may profoundly favor re-epithelialization and reduce inflammation, respectively. Therefore, the P_E_E_NP_PCH containing both components enabled an improved wound healing efficacy, including faster wound closure, efficient re-epithelialization, less inflammation, sufficient collagen deposition, and rapid tissue maturation, as illustrated in Figure 6 and Figure 7. To elucidate the detailed mechanism of how growth factors (e.g., EGF) and enhanced oxygen delivery influence the wound healing process, more studies are certainly needed, and efforts are currently in progress.

## 4. Conclusions

In this study, we successfully developed a new type of chitosan/PVA hetero-composite hydrogel consisting of PHMB, PEs, and ENPs named P_E_E_NP_PCH and demonstrated its therapeutic effects on wound treatment in diabetic rats. The P_E_E_NP_PCH was characterized as an ionic strength-sensitive biomaterial and can offer multiple functions, including antibacterial activity, anti-inflammation, and promotive cell growth, which were verified through in vitro analyses. Most importantly, unlike most of dressings that enhanced air/oxygen delivery was merely achieved by increasing the porosity of the gel matrix, the P_E_E_NP_PCH can proactively offer oxygen delivery through the incorporation of PFC and, therefore, is theoretically able to alleviate hypoxia conditions on diabetic wounds. Based on the results of the animal study using diabetic rats, we further showed that the P_E_E_NP_PCH was indeed able to provide a greater wound closure efficiency, lower inflammatory response, faster deposition of collagen, and rapid recovery of the integrity and functionality of newly formed tissues in vivo compared to the treatments with gauze and/or the commercial dressing HeraDerm. Given the aforementioned effectiveness together with known merits of hydrogel such as moisture maintenance, exceptional hydration capacity, and less adhesiveness, we anticipate that the developed P_E_E_NP_PCH is highly potential for use in clinical diabetic/chronic wound treatment.

## Data Availability

All available data are reported in the article.
